# New device to support patients with acute respiratory distress: high flow, nebulization and oxygen therapy with automated fi02 titration

**DOI:** 10.1186/2197-425X-3-S1-A276

**Published:** 2015-10-01

**Authors:** F Lellouche, E L'Her, P-A Bouchard, M Delorme, T Elfaramawy, B Gosselin

**Affiliations:** Institut Universitaire de Cardiologie et de Pneumologie de Québec, Lac-Beauport, Canada; CHRU Brest, Brest, France; Institut Universitaire de Cardiologie et de Pneumologie de Québec, Québec, Canada; CHU Bordeaux, Bordeaux, France; Université Laval, Département de Génie Électrique et Informatique, Québec, Canada

## Introduction

Hyperoxia induced hypercapnia has been described more than 60 years ago [1] and first recommendations to avoid hyperoxia in COPD patients were provided more than 50 years ago [2]. Alarm bell was recently raised [3] after the demonstration that high oxygen flows could increase mortality in patients with respiratory distress [4].

## Objectives

To develop a device continuously adjusting Fi02 with high flows of air/oxygen based on the Free02 system that titrates oxygen flow delivered to patients with the aim to maintain a constant oxygenation [5].

## Methods

The Free02 system was modified to allow a mixture of oxygen and air administration with a constant gas flow. The proportion of oxygen is based on an advanced closed loop to maintain a constant Sp02. We compare this new prototype with Optiflow set with minimal Fi02 (35%). Both devices are set at 30L/min, and we plan to recruit 10 healthy subjects'. The experimental conditions are the following: healthy subjects will initially breath room air (5 minutes), followed by 5 minutes of induced hypoxemia (nitrogen administration), and return to initial conditions (breathing room air for 5 minutes). We record Sp02, respiratory rate, heart rate and Fi02 delivered.

## Results

We will present results of this comparison in 10 healthy subjects. Initial data demonstrate the feasibility to deliver variable oxygen flows administered with air at high flows (from 20 to 60L/min). In this study, the flow is maintained constant at 30L/min. During nitrogen administration to the healthy subjects, with the new prototype controlling oxygen/air administration, the oxygen increases (leading to a Fi02 increase) to maintain constant the Sp02 level (set at 94% in this study), and the air flow decreases to maintain constant the total flow (30L/min). After cessation of nitrogen, the oxygen flow (and Fi02) are automatically reduced (Figure). In the first included subjects, with Optiflow. hyperoxia is present during the first and third condition (breathing room air) and hypoxemia occur during nitrogen administration.

**Figure 1:**
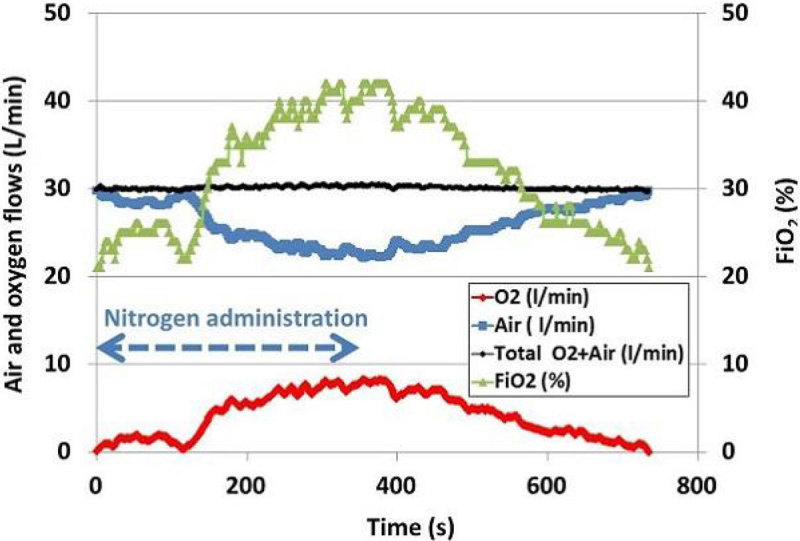
**Automatic adjustment of the Fi02.**

## Conclusions

This new device may help to optimize oxygenation avoiding hypoxemia and hyperoxia during high flow oxygen therapy.

## Grant Acknowledgement

Fond de recherche en Santé du Québec, Canadian Fundation for Innovation
